# Elevated tryptase level in a child with idiopathic anaphylaxis: a case of hereditary alpha-tryptasemia

**DOI:** 10.1186/s13223-023-00858-4

**Published:** 2024-03-27

**Authors:** Vicky Le Blanc, Wade T. A. Watson

**Affiliations:** Division of Allergy, Dalhousie University, IWK Health Centre, Halifax, NS Canada

**Keywords:** Hereditary alpha-tryptasemia, Idiopathic anaphylaxis, Elevated tryptase level

## Abstract

Hereditary alpha-tryptasemia (HαT) is an autosomal dominant disorder estimated to affect 5% of the population. High baseline tryptase level is a consistent finding, but there is a great variability of clinic manifestations, including no symptoms at all. We describe a case of HαT in a 5 years 8 months old girl manifesting with idiopathic anaphylaxis and elevated baseline tryptase level. As more cases of HαT are described, a better understanding of the clinical phenotype will be acquired.

## Introduction

Hereditary alpha-tryptasemia (HαT), caused by increased copies of TPSAB1, is an autosomal dominant disorder affecting approximately 5% of the general population. Elevated baseline tryptase level is a consistent finding among affected families. However, clinical features differ greatly among individuals. While some are asymptomatic, others complain of functional gastrointestinal symptoms, recurrent cutaneous manifestations or constitutional symptoms [1]. HαT has also been associated with increased severity of venom anaphylaxis, systemic mastocytosis (12%) and idiopathic anaphylaxis (17%) [2]. Uncertainty remains around the clinical phenotype of HαT. Describing a case of HαT contributes to better define this unfamiliar disease.

## Discussion

We describe a 5 years 8 months old girl with 2 episodes within 3 months of anaphylaxis manifested by cutaneous (urticaria, angioedema, pruritus) and gastrointestinal symptoms (moderate abdominal pain, emesis, diarrhea). First episode occurred after swimming for one hour and the second occurred 48 h after an acute febrile illness. The first episode required 2 doses of epinephrine for symptoms to resolve. Despite a detailed history, no allergic trigger could be identified. Tryptase level measured during the second acute episode was markedly elevated at 32 μg/L. Baseline tryptase levels were then measured on two separate occasions at 22.7 and 20.6 μg/L. She had no other complaints, no chronic symptoms and no personal history of atopy. Physical examination revealed no sign of lymphadenopathy or hepatosplenomegaly. One small hyperpigmented area was identified on her left leg, but Darier’s sign was negative. Complete blood count and differential, liver enzymes and renal function were normal. Abdominal ultrasound was unremarkable. Genetic testing for KIT mutation was negative. Genetic testing for HαT was not covered in Canada. Therefore, a baseline serum tryptase level was measured in both parents to confirm the diagnosis of HαT. Her mother had a normal level of 7 μg/L and her father had an elevated level of 17 μg/L. He had no symptoms suggestive of HαT and only a personal history of mild atopic dermatitis. Parents desired to screen the patient siblings. One of her sibling had a normal level of 3.2 μg/L and the other had an elevated level of 15.1 μg/L. Both siblings were asymptomatic, with no history of atopy. 14 months later, she had another episode presenting with one hive, pruritus, moderate abdominal pain, diarrhea and nausea. She was treated with a standard dose of second generation antihistamine and symptoms resolved within 5 h. Patient was afebrile at the time of onset, but this last episode occurred approximately 48 h before onset of a viral illness. An epinephrine auto-injector continues to be carried at all time. Prophylaxis treatment with second generation anti-histamine was discussed, but was declined by the family. Absence of treatment was considered appropriate given the lack of chronic symptoms and the frequency of episodes.

## Conclusion

HαT is a condition with a great spectrum of clinical manifestations. Screening family members confirm the diagnosis as it an autosomal dominant disorder. However, screening is controversial as uncertainty remains around the clinical signification of an elevated tryptase level in an asymptomatic individual. Shared decision making as whether to screen other family members or not is certainly warranted. As more cases are described, better understanding of the disease will be acquired.

## Data Availability

Not applicable.

## References

[1] Lyons JJ (2018). Hereditary alpha tryptasemia genotyping and associated clinical features. Immunol Allergy Clin North Am.

[2] Lyons JJ, Chovanec J, O’Connell MP (2021). Heritable risk for severe anaphylaxis associated with increased α-tryptase-encoding germline copy number at TPSAB1. J Allergy Clin Immunol.

